# Changes in Day/Night Activity in the 6-OHDA-Induced Experimental Model of Parkinson’s Disease: Exploring Prodromal Biomarkers

**DOI:** 10.3389/fnins.2020.590029

**Published:** 2020-10-14

**Authors:** Catalina Requejo, Karmele López-de-Ipiña, José Ángel Ruiz-Ortega, Elsa Fernández, Pilar M. Calvo, Teresa Morera-Herreras, Cristina Miguelez, Laura Cardona-Grifoll, Hodei Cepeda, Luisa Ugedo, José Vicente Lafuente

**Affiliations:** ^1^LaNCE, Department of Neuroscience, University of the Basque Country (UPV/EHU), Leioa, Spain; ^2^Department of Neurology, Icahn School of Medicine at Mount Sinai, The Friedman Brain Institute, New York, NY, United States; ^3^EleKin Research Group, Department of Systems Engineering and Automation, University of the Basque Country (UPV/EHU), Donostia, Spain; ^4^Department of Psychiatry, University of Cambridge, Cambridge, United Kingdom; ^5^Department of Pharmacology, University of the Basque Country (UPV/EHU), Leioa, Spain; ^6^Autonomic and Movement Disorders Unit, Neurodegenerative diseases, Biocruces Health Research Institute, Barakaldo, Spain

**Keywords:** 6-hydroxydopamine, circadian rhythms, home-cage monitoring system, prodromal biomarkers, prodromal Parkinson’s disease symptoms, behavior, non-motor deficits, rat

## Abstract

The search for experimental models mimicking an early stage of Parkinson’s disease (PD) before motor manifestations is fundamental in order to explore early signs and get a better prognosis. Interestingly, our previous studies have indicated that 6-hydroxydopamine (6-OHDA) is a suitable model to induce an early degeneration of the nigrostriatal system without any gross motor impairment. Considering our previous findings, we aim to implement a novel system to monitor rats after intrastriatal injection of 6-OHDA to detect and analyze physiological changes underlying prodromal PD. Twenty male Sprague-Dawley rats were unilaterally injected with 6-OHDA (*n* = 10) or saline solution (*n* = 10) into the right striatum and placed in enriched environment cages where the activity was monitored. After 2 weeks, the amphetamine test was performed before the sacrifice. Immunohistochemistry was developed for the morphological evaluation and western blot analysis to assess molecular changes. Home-cage monitoring revealed behavioral changes in response to 6-OHDA administration including significant hyperactivity and hypoactivity during the light and dark phase, respectively, turning out in a change of the circadian timing. A preclinical stage of PD was functionally confirmed with the amphetamine test. Moreover, the loss of tyrosine hydroxylase expression was significantly correlated with the motor results, and 6-OHDA induced early proapoptotic events. Our findings provide evidence for a novel prodromal 6-OHDA model following a customized monitoring system that could give insights to detect non-motor deficits and molecular targets to test neuroprotective/neurorestorative agents.

## Introduction

Parkinson’s disease (PD) is a complex neurologic disorder in which not only motor impairments occur, but also other non-motor symptoms (NMS) play a relevant role, including hyposmia, sleep disorders, depression, constipation and cognitive deficit (Schapira and Tolosa, 2010; Mantri et al., 2018; Weintraub et al., 2018). Most of them often appear earlier than the motor symptomatology, during the so-called prodromal stage and worsen following the disease’s progression (Schapira et al., 2017). Indeed, NMS may manifest several years prior to the onset of motor symptoms, even up to 20 years before the diagnosis, and the prevalence can vary between the patients (Schapira et al., 2017; Rees et al., 2019). In this context, although there are not specific NMS for PD, the presence and combination of various NMS as well as the correlation with an early dopamine depletion may be useful for early diagnosis (Berg et al., 2013; Schapira et al., 2017). Remarkably, slight motor symptoms (<40–60% of dopaminergic neuronal cell loss) have been also associated to the prodromal stage when they do not meet the criteria for the clinical diagnosis of PD (Mahlknecht et al., 2015). Thus, it is essential to establish a correlation between the early manifestations of PD (prodromal stage) with the clinical and pathological stage for an early diagnosis of PD (Mahlknecht et al., 2015; Liepelt-Scarfone et al., 2017; Sherbaf et al., 2018).

Thus, studies about early events of the disease are emerging in order to find some biomarker for an early-premotor diagnosis (Miller and O’Callaghan, 2015). However, the neuroanatomical and etiological background of NMS in PD remain to be elucidated (Schapira et al., 2017). One of the first NMS that is suffered by a high percentage of the patients is the sleep disorder, such as sleep fragmentation, excessive daytime sleepiness or “REM behavior disorder” (RBD), which is a pathology based on increasing muscle tone during the REM (rapid eye movement) sleep (De Lazzari et al., 2018; De Castro Medeiros et al., 2019). Among the mechanisms underlying sleep disturbance, the disruption in the circadian system is attributed as triggering factor (Willison et al., 2013). Indeed, growing evidences support an association between circadian disruption and PD, suggesting that the dopamine depletion may lead to circadian rhythm irregularities including the alteration of the circadian control of the rest/activity rhythms (Tanaka et al., 2012; De Lazzari et al., 2018).

In the search for preclinical models of PD, there is a main challenge for reproducing those functional and pathological changes that could provide the scaffold to find out about the target for the design of neuroprotective therapies before the progression of the disease (Duty and Jenner, 2011; Ko and Bezard, 2017; Lafuente et al., 2017; Iarkov et al., 2020). In this context, the well-known model of the 6-hydroxydopamine (6-OHDA) which is widely used to study motor deficits, can also be useful for identifying non-motor and early motor impairments specially when combined with advanced technological devices (Grandi et al., 2018). In this model, the site of administration and time elapsed after the injection are critical for determining the extent and time course of the lesion (Kirik et al., 2000; Deumens et al., 2002). In our hands, this model is also useful for studying the preclinical phase of PD, as intrastriatal injection of 6-OHDA in adult male rats induced an early degeneration of the nigrostriatal system without any gross motor impairment as well as upregulation of caspase-3 and downregulation of survival signaling pathways (Requejo et al., 2018a,b).

Therefore, in the present study we aimed to detect prodromal changes in the behavior following an experimental model of PD based on the intrastriatal injection of 6-OHDA with a short time of evolution (2 weeks) in adult male rats housed in monitored enriched environment (EE) cages.

## Materials and Methods

### Experimental Design and Housing Conditions

**FIGURE 1:**
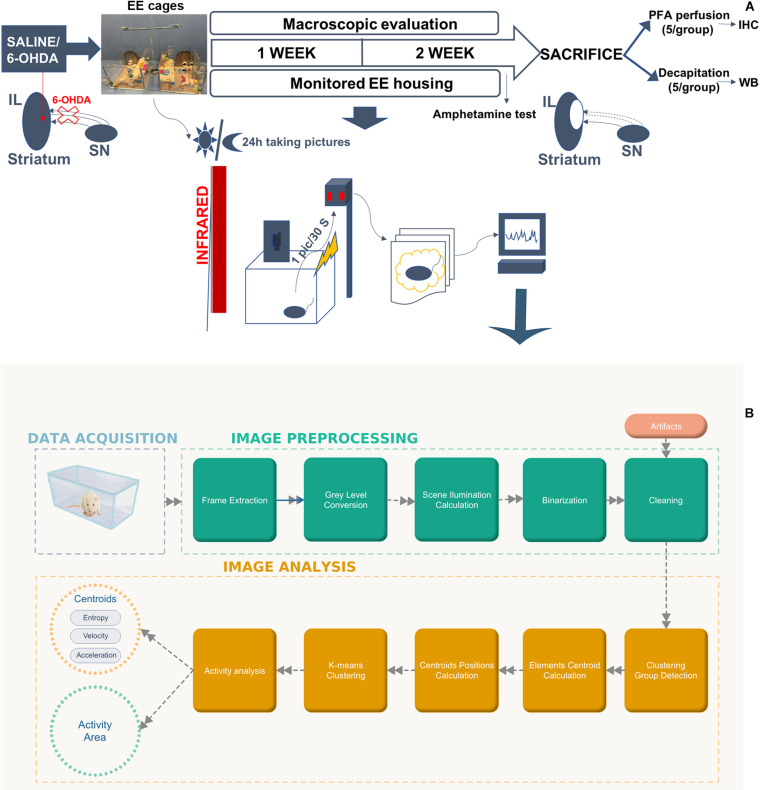
Experimental design and monitoring analysis. **(A)** Schematic representation illustrating the timeline of the experiments. Rats received 6-OHDA (6-OHDA group) or saline solution (Sham group) injections into the right striatum, and immediately after the lesion, they were placed in monitored EE cages for 2 weeks. These EE cages were supplied with an automatic recording system that collect pictures of the EE cages every 30 s under a regular 12 h:12 h light/dark cycle in order to detect activity changes during the light and dark phases. Infrared lighting was also set up to take clear pictures during the dark phase. Two weeks later, the amphetamine test was performed to assess motor symptoms, and then, rats were transcardially perfused or decapitated for the histological and biochemical analysis, respectively. **(B)** Data acquisition, pre-processing and analysis workflow. The process was divided in two phases. (1) Image pre-processing (in green): frame difference, gray level conversion, noise smoothing and binarization). (2) Image analysis (in orange): clustering analysis and centroid calculation, activity analysis. 6-OHDA, 6-hydroxydopamine; EE, enriched environment.

Twenty adult (3-month-old) male Sprague-Dawley rats, weighting 280–300 g at the time of surgery, were obtained from Harlan Laboratories, S, A, (Barcelona, Spain). Rats were randomly assigned to the following groups: Sham group (saline-lesioned rats, *n* = 10) as control and 6-OHDA group (6-OHDA-lesioned rats, *n* = 10). After stereotaxic injection of 6-OHDA or saline solution, the animals were housed for 2 weeks in monitored enriched environment cages (10 animals per cage) consisting of large cage (790 mm × 460 mm × 640 mm) with two floors, which were connected by a plastic ramp and an external running wheel (Figure 1A and Supplementary Figure S1). A 12 h light/12 h dark cycle was established with access to food and water *ad libitum*. The EE cages were developed by our research group and they were designed to host up to 12 rats as well as housing food feeders, water feeding bottles, two semi-closed rooms, a wide-open area, and a ramp to access a runway situated on the upper floor. In addition to this, these EE cages also provide four gates that may be opened to connect to other cages, runways, or exercise wheels. Current EE cages were supplied with an automatic recording system. This novel system consists, basically, of an infrared camera situated above every cage and connected to a raspberry device programmed to collect pictures accurately every 30 s (Figure 1A). In fact, a Raspberry Pi (Raspberry Pi 2 Model B, Raspberry Pi products, United Kingdom) is a standalone embedded computer that measures the size of a credit card. It has enough power to run a linux operating system and connect it to several external devices and sensors. In this case, it was connected to Pi NoIR Camera V2 (Raspberry Pi products, United Kingdom) that provided a clear image of the cage even at night’s dark due to the infrared lighting that was set up throughout the room (Supplementary Figure S1). This in-house customized automatic system continuously recorded a picture every 30 s and saved it in an SD Card. At the end of the experiment, more than 60 gigabytes in pictures were collected and processed in order to measure changes in the activity (Figure 1B).

Two weeks after the intrastriatal injection the amphetamine test was performed for evaluating motor deficits and with the purpose of confirming that no rat presented more than 5 turns per minute (tpm) in order to get a preclinical model (Supplementary Table S1). This criterium was established due to the fact that it has previously been shown that rats rotating more than 5 tpm presented a lesion ranging from 50 to 90% which may predict motor impairment (Björklund and Dunnett, 2019). After the amphetamine test, rats were sacrificed by transcardial perfusion for the histological evaluation or by decapitation for the biochemical analysis (Figure 1A).

All the experimental protocols were reviewed and approved by the Ethical Committee and Animal Welfare of the University of the Basque Country (UPV/EHU, CEBA M20/2015/024, approval date 12/28/2015), and in accordance with the European Community Council Directive on “The Protection of Animals Used for Scientific Purposes” (2010/63/EU) and with Spanish Law (RD 53/2013) for the care and use of laboratory animals.

### 6-OHDA Lesions

Rats were lesioned as we previously described (Requejo et al., 2018a,b). Briefly, thirty minutes before surgery, rats were pre-treated with desipramine (25 mg/kg, i.p., Sigma, St. Louis, United States) and pargyline (50 mg/kg, i.p., Sigma, St. Louis, United States) in order to preserve the noradrenergic system and the degradation of the toxin, respectively. Then, rats were deeply anaesthetized with isoflurane inhalation (1.5–2%; Esteve Química, Barcelona, Spain) and placed in the stereotaxic frame (David Kopf^®^ Instruments). 6-OHDA (3 μg/μl, in 0.02% ascorbic acid) or saline solution (0.9% NaCl) was infused using a 10 μl-Hamilton syringe fitted with a 26-gauge needle at a rate of 0.5 μl/min by a single syringe infusion pump (KDS Scientific, MA, United States).

To generate a mild lesion of the nigrostriatal pathway, three injections of 2.5 μl of the 6-OHDA solution (a total volume of 7.5 μl) were administered at three coordinates into the right striatum, relative to the bregma and dura, with the toothbar set at −2.4: anteroposterior (AP) + 1.3 mm, mediolateral (ML) + 2.8 mm, dorsoventral (DV) −4.5 mm; AP −0.2 mm, ML + 3.0 mm, DV −5.0 mm and AP −0.6 mm, ML + 4.0 mm, DV −5.5 mm, according to Paxinos and Watson atlas (Paxinos and Watson, 2013). The needle was left in place for an additional 5 min to allow the toxin to diffuse into the structure, and then it was slowly retracted. 6-OHDA was prepared daily for each surgery session and changed every 2–3 h. The 6-OHDA solution was kept on ice and protected from light during the surgery to avoid oxidation, which would be indicated by a color change (clear to brown-pink color). Control rats received the same volume of saline solution in the same manner. In addition, a short time of evolution (2 weeks) was elapsed before sacrifice.

### Monitoring Analysis

The in-house customized automatic system based on the image processing with the infrared Camera Module v2 (Pi NoIR; Raspberry Pi Products, United Kingdom) was used for the analysis of the rat group behavior. A schematic diagram of the working procedure for image processing is described in Figure 1B.

This process was divided in two phases: image pre-processing and image analysis.

1. In the first phase video sequences were acquired by a high-quality camera and pre-processed by an own toolbox in MATLAB to create series of binary image frames of the rat group activity. The Toolbox includes: (1) function for image processing, (2) Activity analysis (centroid analysis), and (3) Period analysis among others. Thus, the activity areas (motion detection) were calculated based on a frame difference method (Manchanda and Sharma, 2016; Sengar and Mukhopadhyay, 2016). Frame absolute difference was calculated between two consecutive frames with a sampling period of 30 s. Images were converted from Red Green Blue (RGB) which is a standard format for color image, into gray scale, after that in binary images and then by a threshold filter extracted the main activity area of the system (rats, and objects moved by the rats). Finally, activity areas were defined applying morphological filters to reduce the noise and for smoothing the images. In particular, morphological filters were applied using MATLAB function bwmorph:

Bwmorph (Ir,’open’);% Remove peaks

Bwmorph (Ir,’close’);% Remove hole

2. During the second phase the trajectories of the animal group is generated by the system centroid (the average position of all the activity areas in the binary image) evolution. The centroid is estimated by *k*-means algorithm over the binarized frame series (Eguiraun et al., 2018; López-de-Ipiña et al., 2019). Thus, within each frame, the coordinates of the centers of every object were calculated, and K-means were applied to find the center of the entire group, that is the “centroid.” A centroid with two coordinates:

$$C=\left(x_{c},y_{c}\right)$$

3. The group trajectory consists of the evolution of that centroid from the first frame to the last one.

Finally, the rat groups’ behavior was described and modeled by the following parameters:

1. Number of activity areas in the binary images.
2. Shannon entropy of the centroid trajectory. Shannon entropy is a main concept in information theory and is a measure of average uncertainty (information content). Entropy in biosignals gives information about the system evolution and behavior and can be applied to analyze pathological behaviors (Eguiraun et al., 2018; López-de-Ipiña et al., 2019).

Velocity, speed and acceleration defined as:

(1) $$\left\{ \begin{array}{c} v_{c\,x}=\Delta \,x_{c}/\Delta \,t\\ v_{c\,y}=\Delta \,y_{c}/\Delta \,t \end{array}\right.$$

being speed the absolute value of velocity. An acceleration:

(2) $$\left\{ \begin{array}{c} a_{c\,x}=\Delta \,v_{c\,x}/\Delta \,t\\ a_{c\,y}=\Delta \,v_{c\,y}/\Delta \,t \end{array}\right.$$

### Amphetamine-Induced Rotation Test

Two weeks after intrastriatal injection the amphetamine test was developed following the previously described methodology for this behavioral test (Miguelez et al., 2011). Briefly, D-amphetamine (5 mg/kg in 0.9% NaCl; Sigma-Aldrich, St. Louis, United States) was intraperitoneally administered and the animals were placed in an individual circular cage (rotameter). In the present study, we used a higher dose of D-amphetamine in order to get rotational behavior due to the lesion was minor (Björklund and Dunnett, 2019). After 15 min of latency, the total number of full ipsilateral (IL) rotations was recorded during 90 min (Multicounter LE3806; Harvad Apparatus, Holliston, MA, United States) in order to include the entire response period and avoid differences in variability due to the differences in the pharmacokinetics or in the dopamine release kinetics between rats (Miguelez et al., 2011; Björklund and Dunnett, 2019). Data were expressed as the number of turns per minute.

### Morphological Analysis

Morphological evaluation was performed as we previously described (Requejo et al., 2015, 2018a,b).

#### Tissue Processing for Histological Evaluation

After behavioral test, five rats from each group were intraperitoneally anesthetized with chloral hydrate at 20% (Ref: 141,975, Panreac Quimica SA, Barcelona, Spain) and transcardially perfused with 0.9% sodium chloride followed by 4% paraformaldehyde (PFA) in 0.1 M phosphate-buffered saline (PBS) pH 7.4. Brains were removed, post-fixed overnight in the same fixative solution and next transferred into a cryoprotective solution containing 30% sucrose and 0.1M PBS pH 7.4.50 μm serial coronal sections containing striatum and substantia nigra (SN) were obtained with a freezing microtome and collected following the Paxinos and Watson atlas (Paxinos and Watson, 2013) in 0.6% sodium azide in 0.1 M PBS pH 7.4 for storage and further analysis.

#### TH Immunostaining

Tyrosine hydroxylase (TH) immunohistochemical staining was developed on free-floating coronal slices. Briefly, sections were treated with 3% H_2_0_2_ and 10% methanol in potassium phosphate buffered saline (KPBS), preincubated with 5% normal goat serum (NGS) and 1% Triton X-100 in KPBS (KPBS-T) for 1 h at room temperature (RT), and later incubated overnight at 4°C with rabbit polyclonal anti-TH (Ref: AB-152, Millipore; 1:1,000) in KPBS/T containing 5% NGS, followed by incubation with a secondary biotinylated goat anti-rabbit IgG antibody (Ref: BA1000, Vector Laboratories, Burlingame, CA; 1:200) in 2.5% NGS KPBS/T for 2 h. Afterward, all sections were processed with the avidin-biotin-peroxidase complex for 1 h using a commercial kit (Ref. PK-6102, Elite ABC kit, Vector Laboratories, Burlingame, CA) and the reaction was shown by using 3,3-diaminobenzidine (DAB).

Images were visualized, captured and analyzed at 4x and 40x magnification by an Olympus BX-50 photomicroscope.

#### Double Immunofluorescence Staining of Caspase-3 and NeuN

Coronal sections were incubated with rabbit anti-caspase 3 (H-277) (Ref: sc-7148, Santa Cruz Biotechnology Inc., Spain; 1:50) and monoclonal mouse anti-NeuN (Ref: MAB377, Chemicon International, Inc., Spain; 1:100) diluted in 5% bovine serum albumin (BSA) and 0.1% Triton X-100 in PBS overnight at 4°C. Sections were subsequently incubated with secondary antibodies conjugated to Alexa Fluor-488 (Ref: A11029; Invitrogen; 1:400) and Alexa Fluor-568 (Ref: A11036 Invitrogen; 1:400) for 1 h at room temperature in darkness. Immunostained sections were further reincubated with Hoechst for nuclear counterstaining for 10 min, then slices were washed, mounted on glass slides and coverslipped using Vectashield mounting medium for fluorescence (Ref: x-0517; Vector laboratories).

Images were finally analyzed at 20x magnification using Olympus Fluoview FV500 confocal microscope.

#### Stereological Analysis

The unbiased stereological analysis was performed as previously described (Requejo et al., 2015, 2017, 2018a,b) by using a computerized image analysis system (Mercator Image Analysis system, Explora Nova, La Rochelle, France) connected to an Olympus BX-50 photomicroscope. For this purpose, a total of 7–8 sections in a 1: 8 series were analyzed to cover the entire striatum and SN for each animal.

In brief, in order to evaluate the TH-immunoreactivity (ir) in the IL striatum, the volume of the preserved striatum was calculated using the Cavalieri method (Howard and Reed, 2004) available on Mercator image analysis system by delimiting the negative areas and the entire striatum at 4x magnification, and multiplying these measurements by the thickness of the slices and the intersectional distance (Requejo et al., 2015, 2018a,b). Values were expressed as the percentage of TH-ir volume of the IL striatum respect to the total volume of the IL one.

Changes in the density of dopaminergic neurons and axodendritic network (ADN) were evaluated in both hemispheres through quantifying the TH-ir neuronal density in the entire SN and the TH-ir ADN density in the SN reticulata (SNr) using a stereological tool (an optical fractionator) provided by the Mercator image analysis system (West et al., 1991). Once the region of interest was outlined at 4x magnification, probes of 50 × 50 μm separated by 100 μm were launched into the SN. TH-ir neurons and ADN inside the probe, or crossing the right side of the X–Y axis, were counted at 40x magnification in the entire SN and SNr, respectively. Data were expressed as the percentage of TH-ir neurons or ADN presents on the IL side (lesioned side) vs. the contralateral (CL) hemisphere (non-lesioned hemisphere).

The study of the topological distribution was performed following the previously described approach (Requejo et al., 2015, 2017). For each animal, sections containing the striatum and the SN were evaluated at three representative rostro-caudal levels, respectively, according to Paxinos and Watson’s Atlas (Paxinos and Watson, 2013): rostral (bregma + 0.70 mm), middle (bregma, −0.26 mm), and caudal (bregma, −0.80 mm) striatal sections were considered to determine the TH-ir volume; and rostral (bregma, −5.20 mm), middle (bregma, −5.60), and caudal (bregma, −6.04) nigral sections were examined to estimate the TH-ir neuronal and ADN density.

### Biochemical Analysis

#### Western Blotting

After the behavioral test, 5 rats of each group were anesthetized with chloral hydrate at 20% (Ref: 141,975, Panreac Quimica SA, Barcelona, Spain) and decapitated with a rodent guillotine to obtain fresh brain tissue. IL and CL striatum and SN were collected by microdissection and quickly frozen (Chiu et al., 2007). For this purpose, once brains were removed, coronal slices containing the striatum and SN were cut out and subjected to biopsy with a small biopsy needle. Firstly, the cerebellum was separated, and the brain was cut bi-half into the right and left hemisphere, next cut was for removing the olfactory bulb at the level of the optic chiasm, and the fourth cut was at the level of the pituitary stalk, separating the two coronal slices, one of them containing the striatum and the other one the SN. The frontal cortex from the slice containing striatum was removed in the fifth cut, obtaining the striatum. The SN was collected in the sixth, separating the cortex from the slice. Samples were stored at −80°C until the analysis.

Brain slices were manually homogenized (1:20 w/v) in lysis buffer [10 mM phosphate-buffered (PB) (pH 7.4), 5 mM ethylenediaminetetraacetic acid (EDTA), 5 mM ethyleneglycol-bis(2-aminoethylether)-tetraacetic acid (EGTA), 1 mM dithiotreitol (DTT)] containing a protease inhibitor cocktail (Ref: P-8340, Sigma-Aldrich, Spain). Samples were centrifuged (13,000 rpm at 4°C for 15 min) and soluble proteins were recovered in the supernatants and quantified using the Bio-Rad Protein Assay (Ref: 500-0006, Bio-Rad Laboratories SA, Spain) based on Bradford’s method (Bradford, 1976).

As previously described (Requejo et al., 2018a,b), for each sample 20 μg of proteins was loaded into polyacrylamide CRITERION TGX 12% gels (Bio-Rad Laboratories Inc., Spain) for electrophoresis and then transferred to a PVDF membrane in a Trans-Blot Turbo Transfer System (Bio-Rad, United States) for 7 min. Membranes were incubated with the following primary antibodies: rabbit anti-Phospho-protein kinase B (AKT) (Ser 473) (1:1,000), rabbit anti-AKT (1:1,000), rabbit anti-Phospho-p44/42 MAPK (extracellular signal-regulated protein kinases 1 and 2 (ERK ½)] (Thr202/204) (1:1,000), rabbit anti-P44/42 MAPK (ERK 1/2) (1:1,000) (all of them from Cell Signaling Technology Inc., United States), rabbit anti-caspase 3 (H-277) (1:1,000) (Santa Cruz Biotechnology Inc., Spain), rabbit anti-β-Actin (1:2,000) (Sigma-Aldrich, Spain) and rabbit anti-Beta-Tubulin (1:1,000) (Novus Biologicals, United States) at 4°C overnight. Afterward they were incubated with anti-rabbit IgG peroxidase conjugated secondary antibodies (1:2,000) (Sigma-Aldrich, Spain) for 2 h at RT and immunoblots were developed with an enhanced chemiluminescence kit (GE Healthcare Life Science, United Kingdom). The luminescence of the reaction product was detected in a personal scanner, LI-COR C-DiGit (LI-COR, Bonsai Advanced Technologies SL, Spain), and visualized bands were analyzed with Image Studio Lite 4.0 software (LI-COR, Bonsai Advanced Technologies SL, Spain). β-Actin and β-tubulin were used as loading controls.

### Statistical Analysis

All results were expressed as the mean ± SEM (standard error mean). Statistical analysis was developed with GraphPad Prism (v 5; GraphPad Software, Inc., United States) and SPSS Statistics (v 20; IBM Corporation, Armonk, NY, United States). Prior to the analysis, the Shapiro–Wilk test was used to assess the normal distribution of the samples, and Levene’s test was used to determine the homogeneity of variance. Mann-Whitney *U*-test was performed to assess differences between groups and within groups in the monitoring analysis. The ROC (Receiver Operating Characteristics) curve was performed and the AUC (Area Under the Curve) was calculated in order to measure the accuracy of the model which are a metrics for checking classification models (Hanley and McNeil, 1982; Fawcett, 2006), in this case: Sham group and 6-OHDA group. The behavioral data, stereology and densitometry results were analyzed by means of the two-tailed unpaired Student’s *t*-test to compare differences between groups. A one-way analysis of variance (ANOVA) followed by Tukey’s multiple-comparisons test was used to test the differences between rostro-caudal gradients within each experimental group. The correlation was examined by Pearson product-moment correlation coefficient. Values *P* < 0.05 were considered statistically significant.

## Results

### Behavioral Evaluation

**FIGURE 2:**
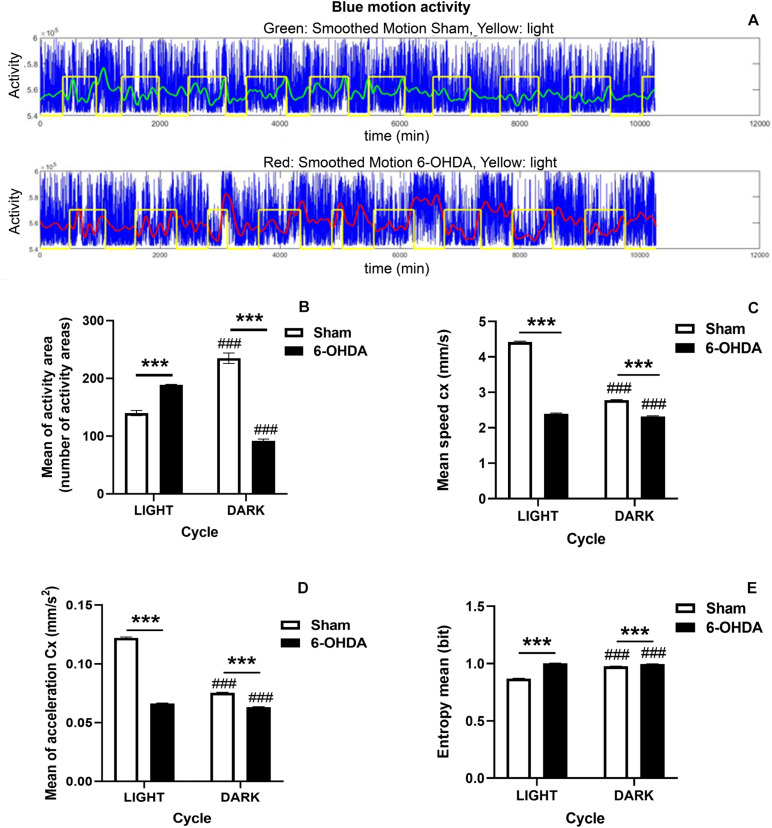
Circadian rhythm disruption in 6-OHDA-induced rats. **(A)** Diagram illustrating the motion activity signal in 6-OHDA (upper panel) and Sham (bottom panel) groups during the whole manuscript. Afterward, the data are preprocessed and selected in order to obtain for the statistical analysis, appropriated balanced datasets of the light and dark periods for the two groups. Blue: motion activity signal, Green: smoothed signal for control group, Red: smoothed signal for 6-OHDA group, Yellow: light level. Graphs depict significant activity changes **(B)**, the centroid features including the mean of centroid speed **(C)**, the mean of centroid acceleration **(D)** and the entropy mean **(E)** between 6-OHDA and Sham groups during the light/dark cycle and within the same group. Data are expressed as mean ± SEM. General analysis was performed by considering 12 h of dark/light periods. Statistical differences appear (*p* < 0.05) for Mann-Whitney *U*-test between both groups and within the same group for both light and dark phases (6-OHDA group vs. Sham group, ***p < 0.001; and light cycle vs. dark cycle within the same group, ^###^*p* < 0.001).

The monitoring of EE cages allows obtaining data about activity and centroid features (speed, acceleration and entropy) during the light/dark cycle. Remarkably, 6-OHDA-lesioned rats decreased significantly the number of activity areas during the dark phase, which is the active period of the rodent circadian cycle, comparing to Sham group (^∗∗∗^*p* < 0.0001, Mann-Whitney *U*-test) (Figure 2B). Moreover, the 6-OHDA group also showed a significant hyperactivity during the light phase (resting period) in comparison with control rats which may be related to a sleep behavior disruption (^∗∗∗^*p* < 0.0001, Mann-Whitney *U*-test; Figures 2A,B). Overall, these results suggested that animals from the 6-OHDA group switched their transitional rhythms respect to the control group. Furthermore, the effect of 6-OHDA on the disruption of circadian rhythms was also supported by a significant difference in the activity between both light and dark phases which was also seen in Sham group (###*p* < 0.0001 activity area mean in light cycle vs. activity area mean in dark phase within the same group, Mann-Whitney *U*-test; Figure 2B).

In line with these results, although Sham group showed more remarkable changes between dark and light cycles in terms of speed and acceleration than 6-OHDA group, entropy was significantly higher in the 6-OHDA group (6-OHDA group vs. Sham group, ^∗∗∗^*p* < 0.0001, Mann-Whitney *U*-test; Figures 2C–E). The fact that entropy was higher supported and confirmed the incipient sleep pathological condition (Figure 2E). Both groups also showed significant differences between both dark and light phases in all the examined parameters related to the centroid evolution indicating different behaviors in both phases according to the light or dark period (###*p* < 0.0001 within the same group, Mann-Whitney *U*-test; Figures 2C–E). Besides, the 6-OHDA effect on the acceleration and speed in both phases pointed to a difficulty in the movement noticed in both phases.

Thus, this is a model that allows integrating information about both dark and light phases.

**FIGURE 3:**
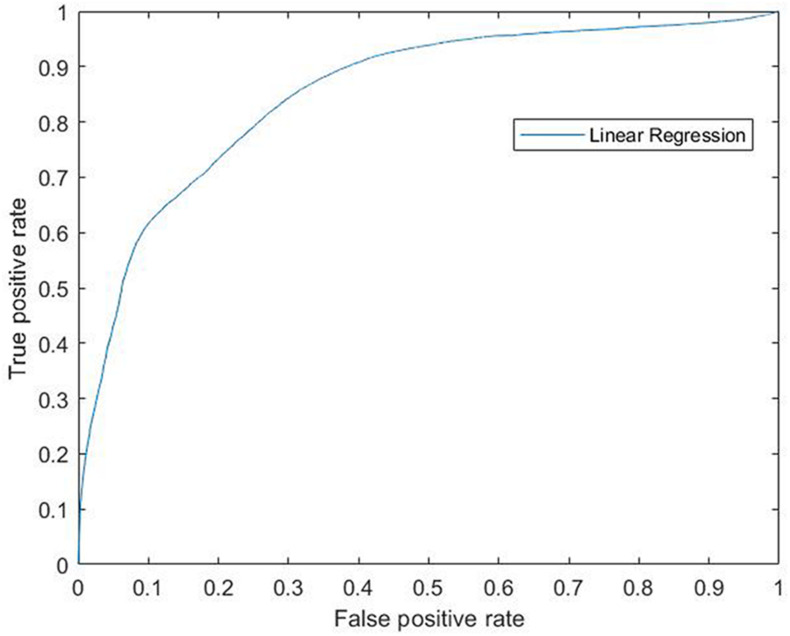
ROC curve for linear regression. Modeling for 6-OHDA and Sham groups. ROC curve (each point on the curve represents a sensitivity/specificity) that reached a significant AUC of 0.854 giving 85% of sensibility and specificity to this model (0.854 ± 0.0047, *p* = 0). ROC, Receiver Operating Characteristics; 6-OHDA, 6-hydroxydopamine; AUC, Area Under the ROC Curve.

Accordingly, the efficiency of this model was confirmed by the results obtained by the ROC curve and its AUC which measure the accuracy of the model by Logistic Regression. In the ROC curve the true positive rate (Sensitivity) is represented in function of the false positive rate (1-Specificity) for different operating points, features of the system, where an AUC of 100% would represent the highest specificity and sensibility of the model to separate both groups. Our data gave a ROC curve (each point on the curve represents a sensitivity/specificity) that reached a significant AUC of 0.854 giving 85% of sensibility and specificity to this model (0.854 ± 0.0047, *p* = 0; Figure 3).

On the other hand, minimal motor deficits were observed in the amphetamine test (Supplementary Table S1). Although animals increased significantly the number of IL turns per minute (tpm) respect to control group, (1.93 ± 0.39 tpm vs. 0.32 ± 0.076 tpm; *t*_(17)_ = 4.292, *p* = 0.0005, Unpaired Student’s *t*-test), no rats turned more than 3.98 turns per minute (0.79–3.98 tpm; Supplementary Figure S2).

Therefore, a prodromal stage would be supported by the mild presence of motor symptoms.

### Morphological Evaluation

Once confirmed the functional changes displayed by 6-OHDA group 2 weeks after 6-OHDA administration, we also assessed whether 6-OHDA exerted a histological effect that we had previously observed 3 weeks after 6-OHDA administration (Requejo et al., 2018a,b).

#### 6-OHDA-Induced Mild Nigrostriatal Dopaminergic Degeneration After a Short Evolution Time

**FIGURE 4:**
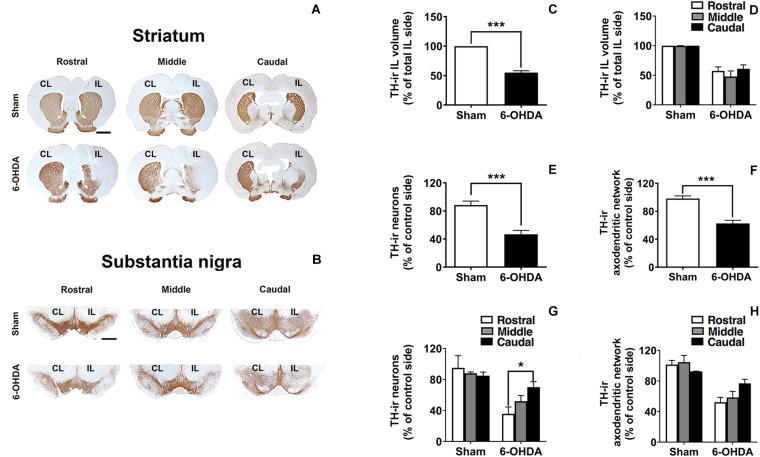
TH-ir loss in the 6-OHDA-induced preclinical model. **(A,B)** Rostro-caudal distribution of coronal sections corresponding to the CL and IL striatum and SN immunostained with tyrosine hydroxylase (TH) in Sham and 6-OHDA groups. **(C,D)** Graphs show the effects of 6-OHDA on the loss of the TH-ir volume in the IL striatum comparing to Sham group (***p < 0.001, Unpaired Student’s *t*-test.) and no significant changes were found along the rostro-caudal axis. **(E,F)** TH-ir neuronal density and TH-ir ADN density decrease significantly following 6-OHDA administration comparing to Sham group in the SN (***p < 0.001, Unpaired Student’s *t*-test). **(G,H)** Topological analysis shows a selective vulnerability in the SN to 6-OHDA decreasing the TH-ir neuronal and the TH-ir ADN density rostro-caudally (*p < 0.05 rostral section vs. caudal section within 6-OHDA group, One-way ANOVA). Data are presented either as the percentage of TH-ir ipsilateral striatal volume respect to the total ipsilateral one **(C,D)** or as the percentage of TH-ir neurons **(E,G)** or TH-ir ADN **(F,H)** remaining in the IL side respect to the CL side. Scale bar: 2 mm **(A)**, 1 mm **(B)**. CL, contralateral; IL, ipsilateral; TH-ir, tyrosine hydroxylase immunoreactivity; 6-OHDA, 6-hydroxydopamine; ADN, axodendritic network; SN, substantia nigra.

Analysis of TH-immunostained sections from the striatum and SN confirmed that, at this survival time, 6-OHDA induced a moderate TH-ir fiber loss either in the striatum or in the SNr (Figure 4). In addition, dopaminergic neurons in the SN were not notably reduced (Figures 4E,G). Rostro-caudal study of TH-ir in the SN indicated that the number of the survival TH-ir neurons as well as the TH-ir axodendritic network (ADN) in 6-OHDA group increased rostro-caudally. Interestingly, one-way ANOVA followed by Turkey *post hoc* test indicated significant differences (*p < 0.05) between rostral and caudal levels within the 6-OHDA group regarding the number of TH-ir neurons (35.61 ± 8.89% of TH-ir neuronal cells at the rostral sections vs. 70.12 ± 7.03% of TH-ir neuronal cells at the caudal sections; *p* = 0.031). However, regarding the number of TH-ir AND no significant differences were found between rostral and caudal sections [52.24 ± 6.32% of TH-ir ADN at the rostral sections vs. 76.86 ± 5.29% of TH-ir of ADN at the caudal section; One-way ANOVA, *F*_(2,9)_ = 3.800, *p* = 0.063; Figures 4G,H]. In addition to this, no topological distribution in the TH-ir striatal fibers was appreciated (Figure 4D).

#### Correlations Between Motor and Morphological Changes

**FIGURE 5:**
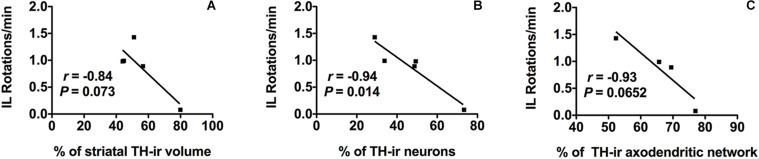
Morpho-functional correlation between amphetamine-induced rotations and dopaminergic neurons in SN in addition to dopaminergic terminals in both striatum and SN after 6-OHDA administration. **(A)** Amphetamine-induced IL rotations correlate negatively with the % of TH-ir striatal volume IL, **(B)** also with the % of TH-ir neuronal density and **(C)** with the % of TH-ir ADN density. Significance. *p* < 0.05, IL rotations vs. TH-ir neurons. SN, substantia nigra; 6-OHDA, 6-hydroxydopamine; TH-ir, tyrosine hydroxylase immunoreactivity; IL, ipsilateral.

The number of IL amphetamine-induced rotations was strongly correlated with the percentage of TH-ir neuronal cell loss (*r* = −0.94) and it was statistically significant (*p* ≤ 0.05) (Figure 5B). On the other hand, the TH-ir terminal loss either in striatum or in SN also showed a correlation with the increase in the number of IL rotations per minute close to be statistically significant (*r* = −0.84, *p* = 0.073 for TH-ir IL striatal volume and *r* = −0.93, *p* = 0.065 for TH-ir AND; Figures 5A,C).

### Biochemical Analysis

**FIGURE 6:**
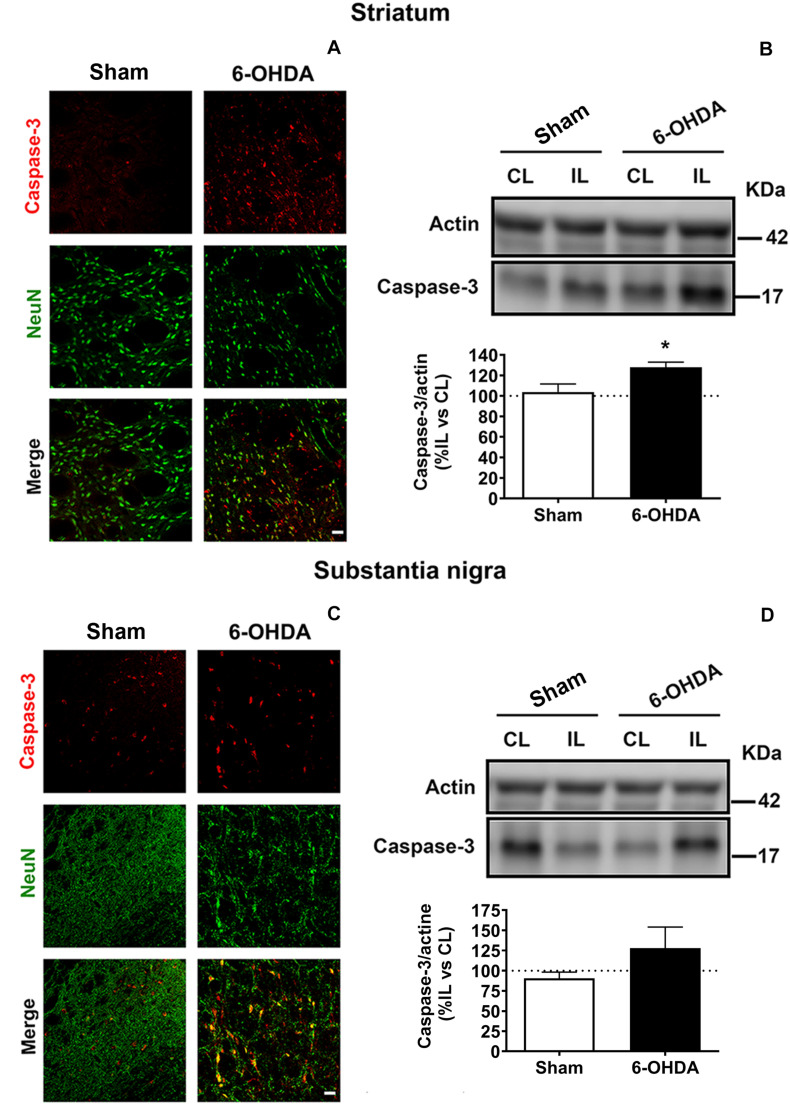
6-OHDA-induced apoptosis is analyzed by immunofluorescence **(A,C)** and western blot **(B,D)** in both striatum and SN. **(A)** Representative immunofluorescent images for caspase-3 (red), NeuN (green) and merge images illustrating the ipsilateral striatum from Sham and 6-OHDA groups. Scale bar = 25 μm. **(B)** Western blot analysis of caspase-3 levels in the striatum reveals a significant increase in 6-OHDA group comparing to Sham group (*p < 0.05, Unpaired Student’s *t*-test). **(C)** Representative immunofluorescent images for caspase-3 (red), NeuN (green) and merge images illustrating the ipsilateral SN from Sham and 6-OHDA groups. Scale bar = 25 μm. **(D)** Western blot analysis of caspase-3 levels in the SN. Actin levels are used as loading control. Densitometric results are normalized with actin and they are set as the % of the caspase-3/actin ratio in the ipsilateral hemisphere respect to the contralateral one. 6-OHDA, 6-hydroxydopamine; SN, substantia nigra.

In order to assess changes in apoptosis, the expression of caspase-3 in neuronal cells was morphologically evaluated by double immunostaining against NeuN (nuclear marker of neuronal cells) and caspase-3 (apoptosis marker). In addition, caspase-3 levels were also biochemically analyzed by western blot in the striatum and SN (Figure 6).

Double immunofluorescence revealed higher caspase-3 immunoreactivity and lower NeuN immunoreactivity in 6-OHDA-lesioned rats compared to Sham group in the IL striatum as well as in the IL SN. In fact, 6-OHDA induced a remarkable apoptosis in neuronal cells due to a considerable amount of NeuN positive cells co-localized with caspase-3 in 6-OHDA group (Figures 6A,C).

Furthermore, immunoblot results were consistent with the outcomes showed by immunostaining. The expression of caspase-3 was upregulated in 6-OHDA-lesioned rats in the striatum and in SN following a similar pattern (127.9 ± 5.1% and 127.9 ± 26.08%, respectively), while caspase-3 activation was barely evidenced in Sham group (103.8 ± 7.6% in the striatum and 90.7 ± 7.57% in the SN). However, statistically significant differences between both groups were only found in the striatum [*t*_(7)_ = 2.426, *p* = 0.045, Unpaired Student’s *t*-test; Figures 6B,D].

However, no significant changes were seen in the pro-survival signaling protein levels (AKT and ERK) between 6-OHDA and Sham groups 2 weeks postinjection, despite both proteins were downregulated in the 6-OHDA group (Supplementary Figure S3).

## Discussion

In the present study, we achieved to monitor the alteration of the circadian cycle in a preclinical model with an automatized and non-invasive method. Indeed, our results evidenced that 2 weeks after the administration of 6-OHDA in the striatum of adult male rats despite inducing only a subclinical motor deficit, other physiological impairments associated with the premotor phase of PD were found. These were related to mild morphological and molecular changes in the dopaminergic system.

Remarkably, our results suggested that 6-OHDA-lesioned rats showed sleep behavioral disruption as an effect of dysfunctional circadian clock. Circadian disruption consists in the alteration at the level of the daily rest/activity rhythm (De Lazzari et al., 2018) and involves to multiple systems, thereby some authors speculate that circadian disfunction can worsen the progression of PD (Willison et al., 2013; Fifel, 2017; De Castro Medeiros et al., 2019). Thus, the use of PD models that reproduce sleep and circadian abnormalities as well as the design of systems that aid to collect data about the evolution of the activity changes in circadian rhythms as we described in this study represent a good strategy for predicting PD prior to the clinical motor deficits manifestation.

In particular, our findings indicated that 6-OHDA-lesioned rats switched their circadian rhythms respect to the control animals reflecting alterations in the temporal patterning of sleep as was previously described in PD patients (Willison et al., 2013). Thus, we can speculate that this change in the pattern of rest/activity seen in response to 6-OHDA may be similar to the insomnia at night or the hypersomnia during the daytime experimented by PD patients (De Castro Medeiros et al., 2019). Behavioral changes related to circadian cycles were already described in the 6-OHDA model, such as alterations on circadian locomotor rhythm parameters (Ben and Bruguerolle, 2000; Gravotta et al., 2011; Masini et al., 2017; De Lazzari et al., 2018; Souza et al., 2018; Ciric et al., 2019; De Castro Medeiros et al., 2019). In fact, some of these studies associated the circadian disruption with the loss of dopaminergic neurons indicating that dopamine may act as a modulator of the circadian system (Isobe and Nishino, 2001; Gravotta et al., 2011; De Lazzari et al., 2018; Souza et al., 2018; Ciric et al., 2019; De Castro Medeiros et al., 2019). Interestingly, our findings are in agreement with these studies, however, we detect changes in both light and dark phases on a regular 12:12 light-dark cycle during 2 weeks after unilateral striatal 6-OHDA lesion. Thus, we also suggest that the early alteration on the circadian rhythms may help to predict the dopamine depletion. In line with our results, Sakata and collaborators found alterations in the sleep behavior in both, light and dark phases. They reported after bilateral injection of 6-OHDA into the ventral tegmental area (VTA) a decrease in REM sleep during the light phase contrary to the dark phase, in which they observed an increase in REM sleep and in non-rapid eye movement (NREM) as well as a reduction in spontaneous activity (Sakata et al., 2002).

The tendency to decrease the activity during the dark phase in response to 6-OHDA was also in accordance with other studies, some of them suggested that 6-OHDA-lesioned rats may show slightly motor impairment and more diurnal activity during the first week after 6-OHDA administration into the SN (Souza et al., 2018). While other studies did not detect activity change during the light phase (Masini et al., 2017).

In addition, 6-OHDA-induced activity changes were also supported by the reduction of speed and acceleration during the light/dark cycle even in the light phase where 6-OHDA-lesioned rats were more active than control rats. These findings may indicate that 6-OHDA-lesioned animals started to present difficulty in the movement. Thus, the assumption of the scarcely motor symptoms as a consequence of circadian disruption reinforces our hypothesis about 6-OHDA enables to induce mild motor symptoms in this model and although they are almost undetectable by conditional motor test. Early motor impairment might be detected by a sophisticated continuous monitoring system like the system we propose.

On the other hand, due to Shannon entropy is often considered as one of the classic and most natural way to measure the expected value (average) of the information in a dysfunctional signal (Eguiraun et al., 2018; López-de-Ipiña et al., 2019), the increase of entropy exhibited by 6-OHDA-lesioned rats confirmed the incipient sleep behavioral disorder.

Moreover, the reliability of this model was also confirmed by the high sensibility and specificity that we found allowing us the correct screening of both animal groups. Taken altogether, we propose the sleep disruption as a promising biomarker to be addressed using the 6-OHDA model in the reported conditions.

Concerning the motor evaluation developed with amphetamine test, the mild increased number of IL rotations was related to a mild degree of denervation in the dorsal subregion of the striatum and with the subsequent mild loss of dopaminergic terminals and neuronal cells in the SN. Interestingly, this correlation pointed out to the accurately or sensitivity of the amphetamine test predicting the dopamine decline (Decressac et al., 2012). Moreover, the mild motor deficit displayed by 6-OHDA-lesioned rats may result in the circadian locomotor activity reduction previously reported in the monitoring analysis. On the other hand, motor evaluation of 6-OHDA lesion was only carried out with the amphetamine test due to the apomorphine test, which is based on the subcutaneous administration of apomorphine to test CL rotations, only predicts an extensive lesion (nearly complete dopamine depletion) (Hudson et al., 1993; Dziewczapolski et al., 1996; Deumens et al., 2002; Yuan et al., 2005; Bilbao et al., 2006). In contrast, the amphetamine test detects motor impairments with only 50% neuronal loss allowing us to assess more moderate lesions (Bilbao et al., 2006). Therefore, the amphetamine test is commonly used in the 6-0HDA model to monitor motor changes and predict the degree of the lesion (Björklund and Dunnett, 2019).

The loss of dopaminergic neurons or fibers found in this model was around 45–55%, indicating that the degree of lesion was lower than the results reported 3 weeks after lesion (Requejo et al., 2018a,b). Thus, the dopamine depletion did not reach the required threshold to develop motor symptoms (Deumens et al., 2002; Padmanabhan and Burke, 2018). However, this loss of dopamine could be sufficient to induce disruption of the circadian rhythms contrary to other studies which suggested a higher dopaminergic neuronal loss (about 60%) in order to detect disruption of circadian locomotor (Souza et al., 2018). In addition, results indicated that a selective vulnerability to the toxin could be observed, pointing out to a dopamine decline topologically distributed in the SN, in concordance with our previous works (Requejo et al., 2015, 2017). In fact, most of the dopaminergic neurons and dopaminergic terminals remained in the caudal axis, decreasing the survival toward rostral axis.

Regarding the mechanisms may underlay the early 6-OHDA-induced neurodegenerative effect on the dopaminergic system; we tested the effect of this toxin over apoptotic cascade and the survival signaling pathways in the striatum and SN. The toxicity mediated by 6-OHDA was able to activate the proapoptotic pathway in neurons increasing caspase-3 levels in the striatum and SN. Indeed, the most remarkable outcomes were found in the striatum where the toxin was injected. These findings supported the capacity of 6-OHDA to trigger the activation of caspase-3 for inducing the cell death through the production of ROS leading to the apoptosis in agreement with other studies (Hernandez-Baltazar et al., 2013). Thus, the early reduction of ROS production could be a strategy to avoid proapoptotic events and the subsequent cell death.

Protein kinase B (AKT) signaling pathway as well as extracellular signal-regulated kinase (ERK) activation play a pivotal role in the regulation of cellular survival and in the inhibition of cell death (Smith and Dahodwala, 2014). However, the effect of 6-OHDA 2 weeks after administration was not sufficient to induce remarkable changes either in AKT or ERK expression in the nigrostriatal pathway. However, our previous studies have shown that the activation of both survival pathways were reduced significantly 3 weeks after 6-OHDA administration (Requejo et al., 2018b). Thereby, although proapoptotic events were evident 2 weeks after lesion, additional time will be required in order to detect changes in survival pathways.

Finally, for the present study the use of EE cages was only with the purpose of monitoring the behavior and assessing functional parameters. In fact, we propose a novel tracking system that allows monitoring animals in their home cages automatically detecting and categorizing behaviors without conditioning their regular environment. Interestingly, our observations indicated that monitored EE cages provided novel additional information for screening changes in the circadian function by monitoring parameters of circadian locomotor activity rhythms (activity area, speed and acceleration, among others) automatically during the light/dark cycle. Since there are discrepancies concerning to the parameters and methods for the evaluation of the active state of the animals (De Castro Medeiros et al., 2019), this monitoring system may represent a promising strategy offering a plethora of different activity parameters to assess along with other additional information about the state of the animal during the light/dark cycle.

## Conclusion

In conclusion, we provide evidences about the suitability of the 6-OHDA-induced model reproducing the prodromal sleep disruption and we implemented a novel system to monitor behavior changes providing an optimal system to test different neuroprotective/neuroregenerative strategies.

## Data Availability Statement

The raw data supporting the conclusions of this article will be made available by the authors, without undue reservation, to any qualified researcher.

## Ethics Statement

The animal study was reviewed and approved by the Ethical Committee and Animal Welfare of the University of the Basque Country Country (UPV/EHU, CEBA M20/2015/024).

## Author Contributions

CR designed the studio, performed the experiments, analyzed the results, prepared the figures, and wrote the manuscript. KL-d-I, EF, and PC analyzed the data from the monitored-EE cages and prepared Figures 1–3. JR-O, TM-H, and CM performed the stereotaxic lesions. LC-G contributed to the immunohistochemistry experiments. HC designed the monitored-EE cages. LU designed the studio, procured funding, provided laboratory resources, and reviewed the manuscript. JL conceived and designed the studio, supervised the work, procured funding, reviewed, and corrected the manuscript. All authors approved the final version of this manuscript.

## Conflict of Interest

The authors declare that the research was conducted in the absence of any commercial or financial relationships that could be construed as a potential conflict of interest.

## Abbreviations

ADN: Axodendritic network density
AKT: Protein kinase B
AUC: Area Under the Curve
CL: Contralateral
EE: Enriched environment
ERK: Extracellular signal-regulated kinase
GFAP: Glial fibrillary acidic protein
IL: Ipsilateral
NMS: Non-motor symptoms
6-OHDA: 6-hydroxydopamine
PD: Parkinson’s disease
REM: Rapid eye movement
NREM: Non-rapid eye movement
RBD: Rapid eye movement sleep behavior disorder
ROC: Receiver Operating Characteristics
ROS: Reactive oxygen species
SN: substantia nigra
SNr: Substantia nigra reticulate
TH: Tyrosine hydroxylase
TH-ir: Tyrosine hydroxylase-immunoreactivity
Tpm: Turns per minute
VTA: Ventral tegmental area.
