# Supplementary material for: Changes in Day/Night Activity in the 6-OHDA-Induced Experimental Model of Parkinson’s Disease: Exploring Prodromal Biomarkers
Source: Front Neurosci. 2020 Oct 14;14:590029. doi: 10.3389/fnins.2020.590029 (PMC7591774; doi:10.3389/fnins.2020.590029)
Supplement: Supplementary file 1 [file Data_Sheet_1.docx]

Supplementary Material

# Supplementary tables

**Supplementary Table 1: Amphetamine-induced ipsilateral rotations per minute in sham (n=10) and 6-OHDA-lesioned rats (n=10).** Data are expressed as the number of full turns per minute (min).

| **Rat ID** | **Group** | **Turns/min** |
| --- | --- | --- |
| #1-16 | Sham | 0.20 |
| #2-16 | Sham | 0.72 |
| #6-16 | Sham | 0.27 |
| #7-16 | Sham | 0.51 |
| #8-16 | Sham | 0.16 |
| #9-16 | Sham | 0.64 |
| #17-16 | Sham | 0.14 |
| #18-16 | Sham | 0.38 |
| #19-16 | Sham | 0.04 |
| #20-16 | Sham | 0.09 |
| #3-16 | 6-OHDA | 0.08* |
| #4-16 | 6-OHDA | 3.98 |
| #5-16 | 6-OHDA | 2.48 |
| #10-16 | 6-OHDA | 2.84 |
| #11-16 | 6-OHDA | 0.89 |
| #12-16 | 6-OHDA | 3.02 |
| #13-16 | 6-OHDA | 0.79 |
| #14-16 | 6-OHDA | 1.43 |
| #15-16 | 6-OHDA | 0.98 |
| #16-16 | 6-OHDA | 0.99 |

6-OHDA, 6-hidroxydopamine; *This rat was excluded of the analysis for technical reasons.

# Supplementary Figures

**
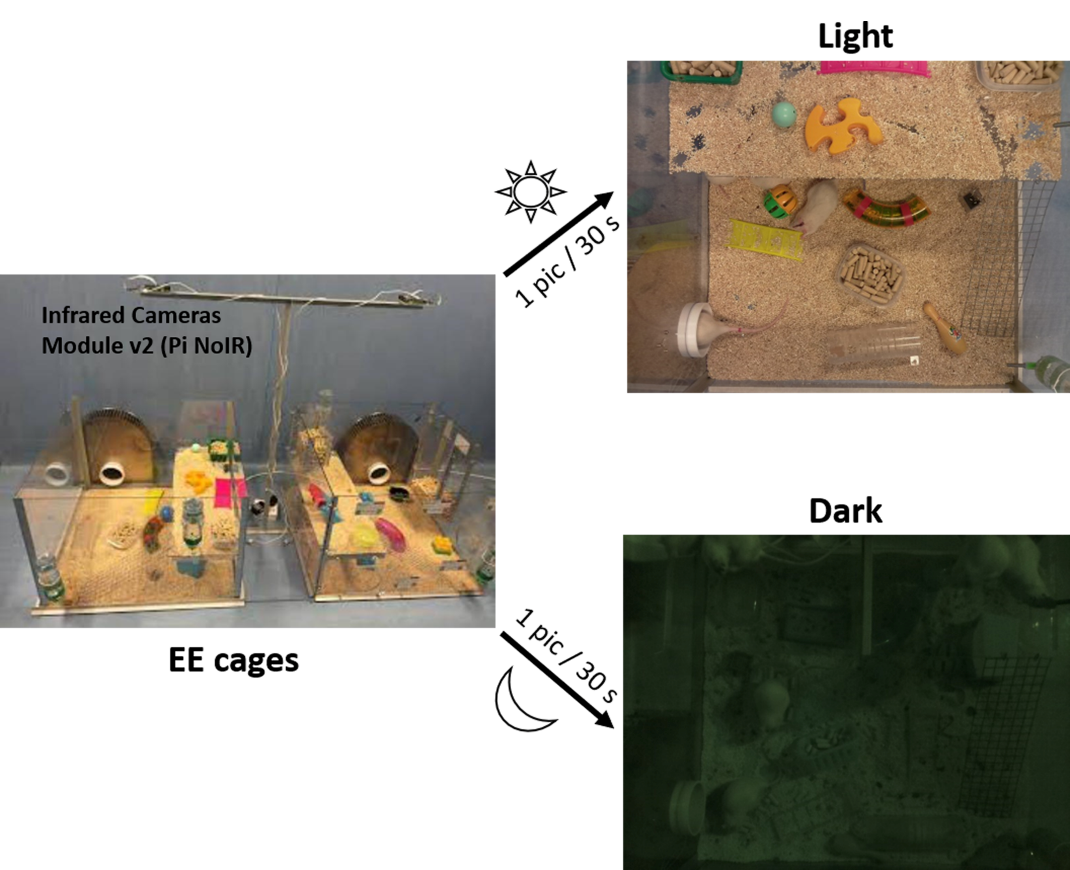
**

**Supplementary Figure 1. Monitored EE cages.** (*Left*) Photograph illustrating the EE cages that consist of large cages with tunnels, toys and two floors connected by a plastic ramp and an external running wheel, and supplied with an additional infrared Camera Module v2 (Pi NoIR). (*Right*) Sample pictures taken by the infrared camera every 30 seconds during the light and dark cycle. EE, enriched environment.


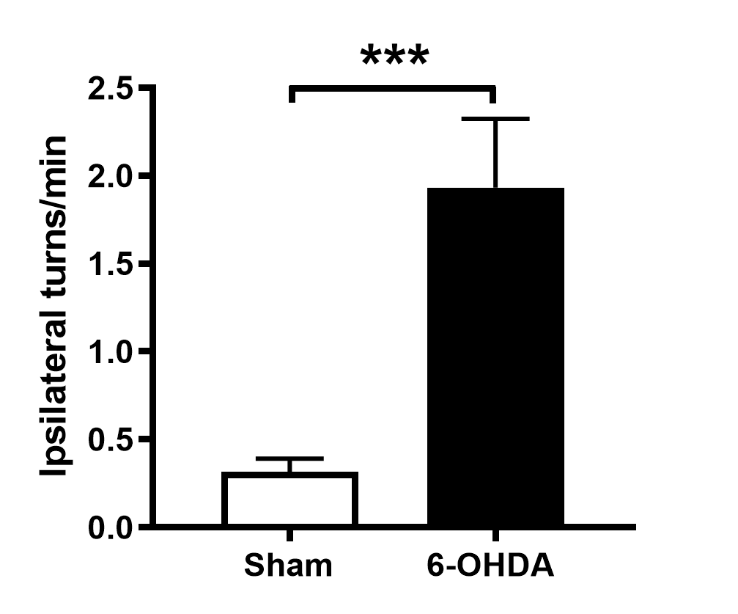


**Supplementary Figure 2.** **Evaluation of motor symptoms.** Graph shows the ipsilateral turns per minute (min) in both groups two weeks after 6-OHDA or saline solution administration. (***p < 0.001, Unpaired Student's t-test).


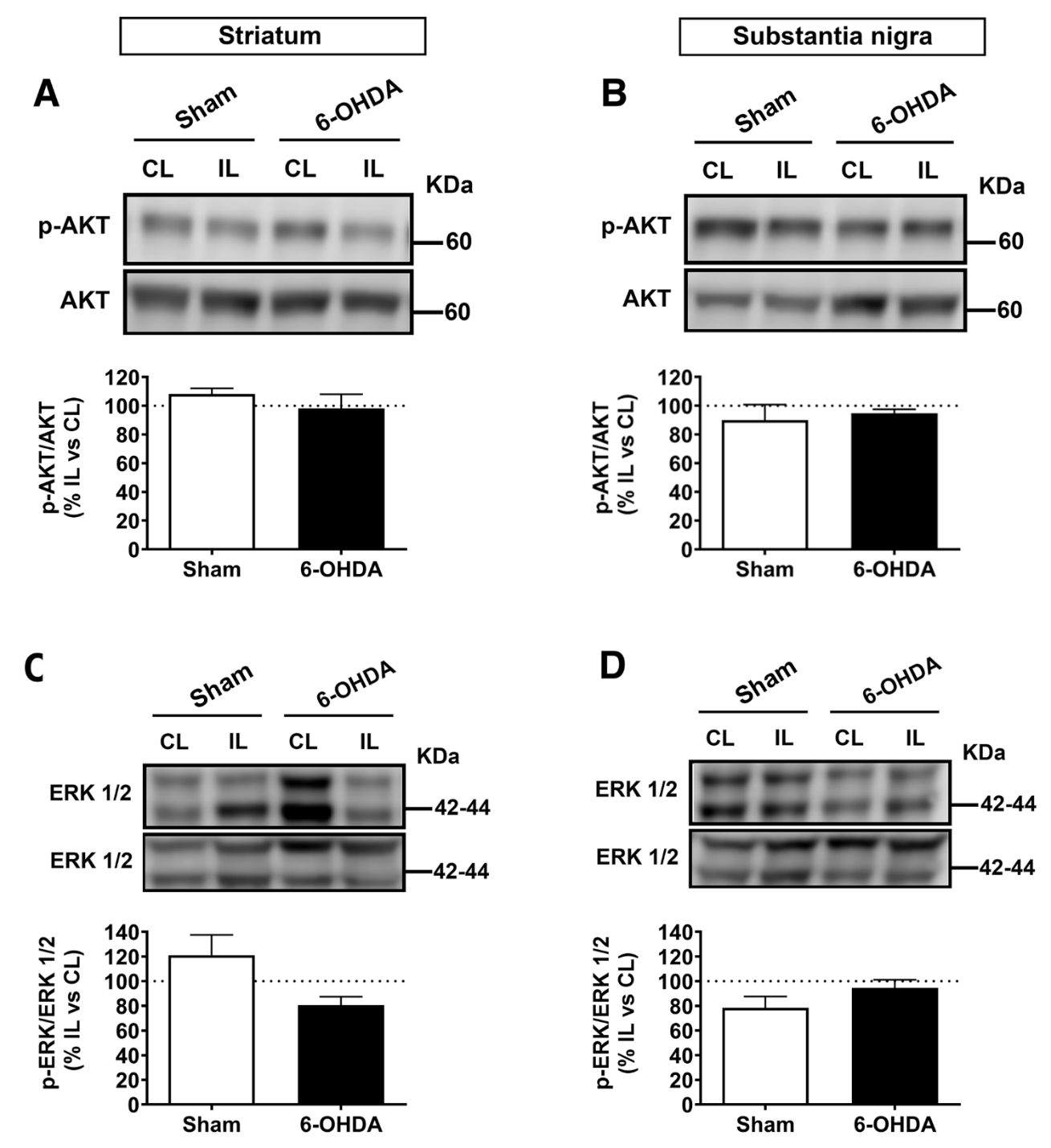


**Supplementary Figure 3. Western blot analysis showing AKT and ERK ½ downregulation after 6-OHDA administration in both stratum and SN.** (A, B) Detection by western blot and quantification of levels of phosphorylated and total forms of AKT in the striatum (A) and SN (B). (C, D) Detection by western blot and quantification of levels of phosphorylated and total forms of ERK ½ in the striatum (C) and SN (D). The phosphorylated form of AKT or ERK ½ was normalized respect to the total form respectively and densitometric results are expressed as the % of p-AKT/AKT or p ERK/ERK 1/2 ratio in the ipsilateral side from the striatum or SN respect to the contralateral one. 6-OHDA, 6-hydroxydopamine; SN, substantia nigra.
